# Schisandrin B Attenuates PM_2.5_-Induced Pyroptosis via Caspase-1 Inhibition and Membrane Repair

**DOI:** 10.3390/membranes16050173

**Published:** 2026-05-09

**Authors:** Le Deng, Lian-Ying Liao, Xiao Ling, Li Li, You-Jie He, Miao-Miao Guo

**Affiliations:** 1 Beijing Key Laboratory of Plant Resources Research and Development, College of Light Industry Science and Engineering, Beijing Technology & Business University, Beijing 102488, China; 2 Beijing Lan Divine Technology Co., Ltd., Beijing 102200, China; 3 College of Biomass Science and Engineering, Sichuan University, Chengdu 610065, China

**Keywords:** cell membrane damage, THP-1, particulate matter 2.5, pyroptosis, schisandrin B

## Abstract

*Schisandra chinensis* (Turcz.) Baill. (Schisandraceae) is a medicinal plant widely distributed in East Asia and has long been used in traditional herbal medicine. Phytochemical studies have identified lignans as the major bioactive constituents of *S. chinensis*, among which Schisandrin B (Sch B) is one of the most abundant and pharmacologically active compounds. Previous studies have demonstrated that Sch B exhibits a variety of biological activities, including antioxidant, anti-inflammatory, hepatoprotective, and cytoprotective effects. As a natural lignan compound derived from *S. chinensis*, Sch B has attracted increasing attention for its potential protective effects against environmental and inflammatory insults. This study aimed to investigate the protective effects of Sch B against PM_2.5_-induced inflammatory injury in THP-1 cells and to elucidate the underlying molecular mechanisms. An in vitro PM_2.5_-induced THP-1 cell injury model was established by stimulating THP-1 cells with PM_2.5_. Subsequently, Sch B was applied to the model, and inflammation-related indicators and pathways were detected using methods such as ELISA, PCR, and Western Blot (WB). The results showed that Sch B significantly inhibited interleukin-1β (IL-1β) secretion and attenuated PM_2.5_-induced pyroptosis in THP-1 cells. Mechanistically, Sch B alleviated cell membrane damage and inflammatory factor release by suppressing Caspase-1 activation and inhibiting the cleavage of gasdermin D (GSDMD) into its active N-terminal fragment (N-GSDMD). Furthermore, Sch B treatment was associated with the up-regulation of ALG-2, ALIX, and TSG101, suggesting the potential involvement of ESCRT-III-associated membrane repair mechanisms. In conclusion, Sch B, a natural lignan compound derived from *S. chinensis*, exhibits protective effects against PM_2.5_-induced THP-1 cell pyroptosis by reducing cell membrane damage and inflammatory cytokine release. These effects are associated with the inhibition of Caspase-1 activity and GSDMD cleavage, as well as the activation of ESCRT-III-associated membrane repair responses. Collectively, these findings highlight the potential of Sch B as a natural cytoprotective compound against particulate matter-induced inflammatory injury.

## 1. Introduction

**Figure 1:**
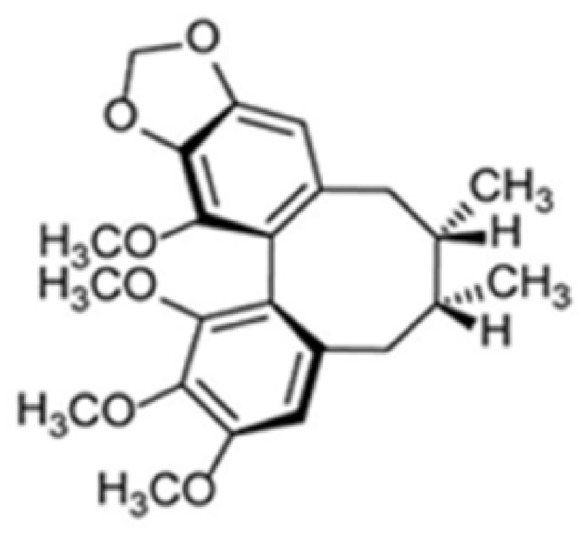
The chemical structure of Schisandrin B (Sch B).

*Schisandra chinensis* (Turcz.) Baill., commonly known as Wuweizi, is a well-known medicinal plant widely used in traditional Chinese medicine and East Asian ethnomedicine. According to ethnopharmacological records and classical herbal texts, *S. chinensis* has long been prescribed for the treatment of insomnia, chronic cough, respiratory disorders, fatigue, and liver dysfunction [1]. Phytochemical studies have identified a series of bioactive lignans from *S. chinensis*, among which schisandrin B (Sch B) is considered one of the major active constituents responsible for its pharmacological activities (its chemical structure is shown in Figure 1). Sch B has been reported to exert antioxidant, anti-inflammatory, anticancer, and neuroprotective effects [2,3,4]. Previous studies have shown that Sch B can inhibit NLRP3 inflammasome activation by reducing intracellular NLRP3 expression and suppressing mitochondrial reactive oxygen species (ROS) production in various injury models [5]. These traditional indications suggest potential anti-inflammatory and cytoprotective activities, which may be relevant to cellular injury induced by environmental pollutants such as PM_2.5_.

Particulate matter 2.5 (PM_2.5_) is a term used to describe particles with an aerodynamic equivalent diameter of less than _2.5_ μm in ambient air, which are often referred to as fine particles. In urban areas, PM_2.5_ pollution from the rapid industrial development, urban traffic, and construction has become a matter of social concern. PM is known to damage the human respiratory and cardiovascular systems via induction of oxidative stress, immune-mediated inflammation, and mutagenic effects, all of which can adversely affect human health [6]. Recent studies have further linked PM_2.5_ exposure to inflammasome-mediated pyroptosis through the ROS/NF-κB/NLRP3F signaling axis, leading to Caspase-1 activation and downstream gasdermin D (GSDMD) cleavage [7].

Pyroptosis is an inflammatory cell death mode, which is considered intermediate between apoptosis and necrosis. The main features of pyroptosis are the formation of pores and vesicles in the cell membrane; cell swelling, rupture, secretion of proinflammatory cytokines (IL-1β and IL-18); and the release of cellular contents to the outside of the cell. Pyroptosis produces proinflammatory signals, rapidly activates the body’s natural immune mechanism, and causes an inflammatory response, which eventually leads to osmotic disintegration of cells [7]. In the classical mechanism, activated Caspase-1 cleaves GSDMD to release its N-terminal domain, which forms large oligomeric pores in the plasma membrane, resulting in the release of IL-1β and IL-18 [8,9].

Crucially, cells can counteract this damage via the ESCRT-mediated membrane repair pathway. Ca^2+^ influx through GSDMD pores triggers the recruitment of ESCRT-III components, including ALG-2, ALIX, and TSG101, to damaged membrane regions to limit pyroptosis and maintain cell integrity [10]. Whether Sch B can modulate this interplay between pore formation and ESCRT-mediated repair remains to be elucidated.

Sch B suppressed the expression of inflammatory factors by inhibiting the c-Jun N-terminal/p38/NF-κB/signaling pathway in the LPS-stimulated RAW 264.7 cell model. Hu et al. [11] established an in vitro model of acute exacerbation of chronic obstructive pulmonary disease (COPD) by treating A549 lung epithelial cells with LPS combined with cigarette smoke extract. Treatment with *Schisandra chinensis* ethyl acetate extract or its major lignan Sch B significantly reduced the mRNA expression and secretion of inflammatory mediators, including IL-8 and COX-2, and decreased NF-κB phosphorylation, thereby improving the inflammatory state of the cells.

Based on its long-standing traditional use in treating chronic inflammatory and respiratory conditions, Sch B, a major bioactive lignan isolated from *S. chinensis*, has attracted attention for its protective effects. Given that PM_2.5_-induced injury is closely associated with the ROS/NLRP3/Caspase-1/GSDMD axis, we hypothesized that Sch B may protect against PM_2.5_-induced damage by modulating this pyroptosis pathway and potentially enhancing ESCRT-mediated membrane repair. Therefore, this study was designed to evaluate the anti-PM_2.5_ activity of Sch B in a THP-1 model, to elucidate its underlying mechanisms with a focus on Caspase-1/GSDMD-mediated pyroptosis and membrane integrity, and to provide evidence supporting the application of Sch B as an anti-pollution and anti-inflammatory agent.

## 2. Materials and Methods

### 2.1. Collection and Processing of PM_2.5_

A smart medium-flow atmospheric particulate matter sampler TH-150H (Wuhan Tianhong Environmental Protection Industry, Wuhan, China) was used to collect PM_2.5_ particles from the air on the Fucheng Road, Haidian District, Beijing (coordinates: 39.93° N, 116.32° E), at an altitude of approximately 40 m above the ground. The air sampler with a flow rate of 100 L/min was fitted with a glass fiber filter membrane to collect the particulates. The membranes were then cut into pieces of 1 cm^2^, which were submerged in 15 mL of ultrapure water, followed by three cycles of ultrasonication for 15 min each at 70 kHz. The samples were then passed through clean gauze, and the filtrate was prefrozen at −20 °C for 4 h and then vacuum freeze-dried for 24 h [7]. The collected PM_2.5_ particles were then centrifuged in 2 mL of pure water at 10,000 rpm at 25 °C three times. The final pellet was resuspended in 2 mL of ultrapure water, evenly pipetted into a clean Petri dish, and prefrozen at −20 °C for 4 h. Ten milligrams of dried PM_2.5_ were sterilized by ultraviolet irradiation for 1 h and dissolved in 1 mL of PBS containing 10% (*v*/*v*) DMSO, followed by three cycles of ultrasonication (15 min each at 70 kHz) to ensure uniform dispersion. The Schisandrin B stock solution was similarly prepared using DMSO as the primary solvent. In all subsequent cell experiments, the final concentration of DMSO in the culture medium was maintained below 0.1% (*v*/*v*). Preliminary experiments were conducted to compare a 0.1% DMSO vehicle control with a blank control group; no significant differences were observed in cell viability or pyroptosis markers, confirming that the solvent at this concentration did not interfere with the experimental results. A 10 mg/mL PM_2.5_ stock solution was stored at −20 °C and diluted with serum-free RPMI 1640 medium for subsequent cell experiments. The morphological characteristics and particle size of the pretreated PM_2.5_ were verified by scanning electron microscopy (SEM) (see Supplementary Figure S1).

Subsequently, these samples were placed on a filter membrane, which was stuck to a sample holder using a conductive tape and then treated with platinum in a GVC-2000 magnetron sputtering apparatus (Kyky Technology, Beijing, China) to prepare a conductive film. Each spray cycle lasted for 100 s, and each sample was sprayed twice [8]. The samples were then adhered to the sample stage of an Apreo 2 field scanning electron microscope (SEM; Thermo Fisher Scientific, Waltham, MA, USA) using a conductive medium and were evaluated under a vacuum at a voltage of 10 kV.

### 2.2. Reagents

Sc B is a lignan metabolite originally isolated from the dried fruit of *Schisandra chinensis* (Turcz.) Baill. [Schisandraceae; Schisandrae chinensis fructus], a medicinal botanical drug widely used in Traditional Chinese Medicine. The botanical name and taxonomic information were validated using Kew Plants of the World Online. Sch B was purchased as an analytical standard (HPLC ≥ 98%, Shanghai Yuanye Bio-Technology Co., Ltd., Shanghai, China, catalog number: B21327). The chemical characterization and reporting of Sch B followed the ConPhyMP guidelines for best practice in pharmacological research [12]. It was dissolved in DMSO to prepare a 10 mM stock solution and stored at −20 °C. The stock solution was diluted with serum-free RPMI 1640 medium for subsequent cell experiments.

### 2.3. Cell Culture

THP-1 cells (human monocytic leukemia cell line; catalog no. TCHu57) from the National Collection of Authenticated Cell Cultures were maintained in RPMI 1640 medium (Thermo Fisher Scientific) supplemented with 10% fetal bovine serum (Thermo Fisher Scientific) and a 1% penicillin–streptomycin solution (Thermo Fisher Scientific). Cells were incubated in a humidified atmosphere of 5% CO_2_ and used in the exponential growth phase in subsequent experiments.

### 2.4. Cell Viability Assay

THP-1 cells were seeded in a 96-well culture plate at 2 × 10^6^ cells/mL in a volume of 100 µL per well, followed by the addition of 100 µL of samples at different concentrations and incubation for 24 h. Subsequently, 10 μL of the Cell Counting Kit-8 reagent (Dojindo, Kumamoto, Japan) was added to each well in the dark, and the plate was incubated at 37 °C for 1 h, followed by the measurement of absorbance at 450 nm.

### 2.5. Establishment of a PM_2.5_-Induced Cell Injury Model in THP-1 Cells

PM_2.5_ stock solution (10 mg/mL) was diluted with serum-free RPMI 1640 medium to the indicated concentrations. THP-1 cells were used directly without PMA-induced differentiation or additional priming agents and were seeded into 24-well plates at a density of 2 × 10^6^ cells/mL (500 μL per well) were exposed to a range of PM_2.5_ concentrations (50, 100, 200, and 400 μg/mL) for 24 h to determine the optimal injury dose. Cells were incubated at 37 °C in a humidified atmosphere containing 5% CO_2_ for 24 h. Based on the results of cell viability and pyroptosis rates, concentrations of 50 and 100 μg/mL were selected for evaluating the protective effects, with 100 μg/mL identified as the optimal concentration for all subsequent mechanistic experiments. The final DMSO concentration in all treatment groups was strictly maintained at 0.1% (*v*/*v*). After incubation, cells were collected and pyroptotic cell death was assessed using an Annexin V-FITC/PI staining kit according to the manufacturer’s instructions [5].

A total of 20,000 events per sample were acquired using a BD Accuri C6 flow cytometer (BD Biosciences, San Jose, CA, USA). To ensure the accuracy of the data, cell debris and doublets were excluded from the analysis by gating on forward scatter (FSC) vs. side scatter (SSC) and FSC-A vs. FSC-H plots, respectively. The quadrants of the Annexin V/PI scatter plots were interpreted as follows: Q4 (Annexin V−/PI−, live cells), Q3 (Annexin V+/PI−, early apoptotic cells), and Q2 (Annexin V+/PI+, pyroptotic or late apoptotic cells). Since gasdermin D-mediated pyroptosis is characterized by rapid plasma membrane rupture and PI influx, the “pyroptosis rate” reported in this study was defined as the percentage of Annexin V+/PI+ double-positive cells (Q2 quadrant).

Based on the preliminary dose–response assays, 100 μg/mL PM_2.5_ was identified as the optimal challenging dose for subsequent mechanistic experiments. At this concentration, PM_2.5_ effectively triggered canonical pyroptotic markers (such as Caspase-1 activation and LDH release) while maintaining sufficient cell viability for observing the protective effects of Sch B, thus striking a balance between cellular stress and experimental sensitivity.

### 2.6. Evaluation of the Inflammatory Response

THP-1 cells were seeded in 96-well plates at 3 × 10^6^ cells/mL. To evaluate the anti-inflammatory effect, cells were pretreated with various concentrations of Sch B (5, 10, or 20 μM) for 4 h, followed by co-exposure to PM_2.5_ (100 μg/mL) for 24 h. For validation of Caspase-1-dependent inflammatory responses, cells were also pretreated with Ac-YVAD-CMK (10 μM proteintech, Rosemont, IL, USA), a selective Caspase-1 inhibitor, for 4 h prior to PM_2.5_ stimulation as a positive control. After 24 h of incubation at 37 °C in a humidified atmosphere of 5% CO_2_, culture supernatants were collected for the analysis of IL-1β levels.

### 2.7. Measurement of Caspase-1 Activity

THP-1 cells were seeded and treated according to the previously established PM_2.5_-induced cell injury model. Cells were collected by low-speed centrifugation (1000× *g*, 5 min, 25 °C) and Caspase-1 activity was measured using the Caspase-Glo^®^ 1 Inflammasome Assay (Promega, Madison, WI, USA) following the manufacturer’s instructions. All samples were incubated at 25 °C for 1 h to stabilize the luminescence signal prior to measurement.

To determine the optimal time point for Caspase-1 activation, preliminary experiments were conducted at 0, 2, 4, 6, and 8 h after PM_2.5_ exposure, and Caspase-1 activity peaked at 4 h. Therefore, subsequent experiments used a 4 h stimulation period.

Experimental groups included:

Blank control: THP-1 cells cultured in serum-free RPMI 1640 medium without PM_2.5_ exposure.

Negative control: THP-1 cells exposed to PM_2.5_ (100 μg/mL) for 4 h.

Sch B treatment groups: THP-1 cells pretreated with Sch B (5, 10, or 20 μM) for 4 h, followed by PM_2.5_ (100 μg/mL) stimulation for 4 h.

Positive control: To confirm the specificity of Caspase-1 activation, cells were pretreated with the irreversible selective inhibitor Ac-YVAD-CMK (10 μM) for 4 h prior to stimulation. Additionally, the reversible inhibitor Ac-YVAD-CHO (provided with the assay kit) was employed as a technical positive control during the Caspase-Glo^®^ 1 reaction to ensure assay specificity.

This experimental design allows for evaluation of the Caspase-1-dependent inflammatory response induced by PM_2.5_ and the inhibitory effect of Sch B, with the positive control confirming the specificity of Caspase-1 involvement.

### 2.8. Quantitative Real-Time Polymerase Chain Reaction (PCR)

THP-1 cells were seeded in 96-well plates at 3 × 10^6^ cells/mL and treated with various concentrations of Sch B for 4 h, followed by exposure to PM_2.5_ at 100 µg/mL for 24 h. Total RNA was extracted from cells using a total RNA extraction kit (Chengdu Forge Biotechnology, Chengdu, China). The extracted RNA was used as a template with the PrimeScript™ RT master mix (Takara, Kusatsu, Japan) and TB Green^®^ Premix Ex Taq™ II (Takara) to quantitatively evaluate the expression of various genes involved in inflammatory responses and pyroptosis (Table 1). The expression value of the reference sample (GAPDH) was set to 1, and the 2^−∆∆Ct^ method was used to calculate the relative target gene expression in each sample.

### 2.9. Western Blotting

THP-1 cells were plated at a density of 3 × 10^6^ cells/mL into a 24-well plate and treated as previously described. The collected cells were incubated with a lysis buffer, and the lysate was centrifuged to remove the cell debris. Protein levels were quantified using a bicinchoninic acid assay kit (Weiao Biotechnology, Shanghai, China), and equal amounts of protein (20–35 µg) were separated using 10% sodium dodecyl sulfate–polyacrylamide gel electrophoresis, followed by transfer onto polyvinylidene difluoride membranes. The membranes were blocked with 5% bovine serum albumin (BSA) in Tris-buffered saline/Tween 20 (TBST; Weiao Biotechnology) for 1 h at 25 °C, followed by incubation with the following primary antibodies at 4 °C overnight: rabbit anti-GSDMD (Cell Signaling Technology, Danvers, MA, USA), rabbit anti-N-terminal GSDMD (Abcam, Cambridge, UK), rabbit anti-ALG-2 (Abcam), mouse anti-TSG101 (Abcam), mouse anti-ALIX (Cell Signaling Technology), rabbit anti-CHMP4B (Cell Signaling Technology), mouse anti-β-actin (Weiao Biotechnology), and mouse anti-GAPDH (Weiao Biotechnology). The primary antibodies were diluted with TBST supplemented with 5% BSA at a ratio of 1:1000, while a horseradish peroxidase-labeled secondary antibody was diluted in TBST at a ratio of 1:2000. Washed membranes were then incubated with the secondary antibody for 1 h at 25 °C, washed again with TBST, and visualized using enhanced chemiluminescence reagents (Weiao Biotechnology) and a chemiluminescence image analysis system (Tanon-5200 Multi; Tanon, Shanghai, China). Using ImageJ (version 1.54p, National Institutes of Health, Bethesda, MD, USA), the gray value of the target protein band on the membrane was obtained and used to calculate the expression level of the target protein relative to that of the internal reference protein.

The membranes were horizontally cropped into strips based on molecular weight markers prior to antibody incubation to optimize the simultaneous detection of multiple proteins. Full-view images of these strips are provided in the Supplementary Information.

### 2.10. Measurement of Intracellular Ca^2+^

THP-1 cells were treated according to the established PM_2.5_-induced cell injury model. Briefly, cells exposed to PM_2.5_ (100 μg/mL) for 24 h served as the negative control, while cells cultured in serum-free RPMI 1640 medium without PM_2.5_ served as the blank control.

To evaluate the effect of Sch B on intracellular Ca^2+^ influx, THP-1 cells were pretreated with Sch B (5 or 10 μM) for 4 h, followed by PM_2.5_ exposure (100 μg/mL) for 24 h. Cells were then collected by centrifugation at 1000 rpm for 5 min at 25 °C and washed twice with Hank’s balanced salt solution (HBSS).

Cells were incubated with 5 μmol/L Fluo-4 AM at 37 °C for 30 min in the dark, washed with HBSS, and further incubated at 37 °C for 30 min to allow for complete de-esterification. Intracellular Ca^2+^ fluorescence was analyzed using the FL-1 channel of a BD Accuri C6 flow cytometer.

### 2.11. Data and Statistical Analysis

All data are expressed as the mean ± standard deviation from at least three independent experiments. Statistical significance of differences among the groups was evaluated using one-way analysis of variance and Duncan’s multiple range test. A value of *p* < 0.05 was considered statistically significant. All statistical analyses were performed using the SPSS version 25 software (IBM Corp., New York, NY, USA).

## 3. Results

### 3.1. Effect of PM_2.5_ on THP-1 Cell Pyroptosis Rate Measured by Flow Cytometry

**Figure 2:**
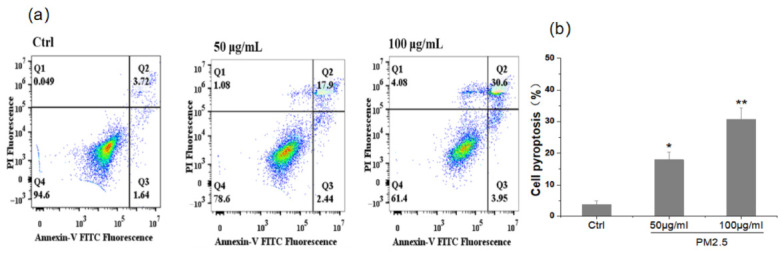
Pro-pyroptotic effect of PM_2.5_ on THP-1 cells. (**a**) Representative flow cytometric scatter plots of THP-1 cells stained with Annexin V-FITC and PI. The quadrant gates are defined as: Q4 (live cells), Q3 (early apoptotic cells), and Q2 (pyroptotic cells). (**b**) Quantitative analysis of the cell pyroptosis rate (defined as the percentage of cells in the Q2 quadrant). Data are presented as mean ± SD (*n* = 3). * *p* < 0.05, ** *p* < 0.01 vs. the control group.

The THP-1 cells were stimulated with concentrations of 50 and 100 μg/mL PM_2.5_ for 24 h, respectively, and Flow cytometric analysis (Figure 2a) demonstrated a concentration-dependent increase in the Annexin V+/PI+ cell population (Q2 quadrant). Specifically, the percentage of cells in the Q2 quadrant significantly rose from 3.72% in the control group to 17.9% and 30.6% in the 50 and 100 μg/mL PM_2.5_-treated groups, respectively (Figure 2b). While the PI-positive region can include various forms of membrane rupture, we identified the Annexin V+/PI+ (Q2) population as representing pyroptotic cells in this study, which was further validated by the detection of GSDMD cleavage and Caspase-1 activation in subsequent experiments (Figure 2).

### 3.2. Effect of Sch B on PM_2.5_-Induced Cell Membrane Damage

#### 3.2.1. Sch B Mitigates PM_2.5_-Induced Increase in IL-1β Release

**Figure 3:**
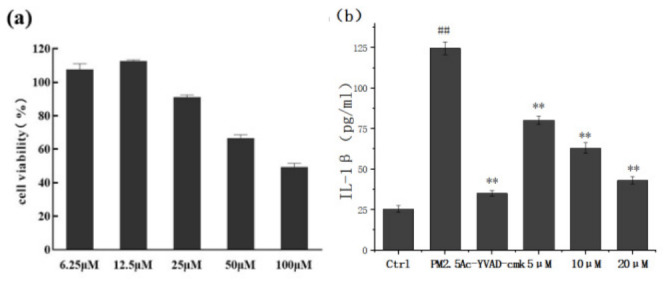
The effect of Sch B on PM_2.5_-induced inflammation. (**a**) The cell viability of THP-1 cells treated with different concentrations of Sch B. (**b**) Effect of Sch B on PM_2.5_-induced IL-1β release in THP-1 cells under PM_2.5_ stimulation (** *p* < 0.01 vs. the group treated with 100 μg/mL PM_2.5_; ## *p* < 0.01 vs. control).

The effect of Sch B on PM_2.5_-induced cell membrane damage was evaluated based on the release of IL-1β, the activity of Caspase-1, and the expression of GSDMD. When exploring the safe concentration of Sch B for THP-1 cells, it was found that the cell survival rate was greater than 80% in the concentration range of 0–25 μM Sch B (Figure 3a). To further investigate its protective effects, Sch B at concentrations of 5, 10, and 20 μM were employed. In all treatment groups, the final concentration of DMSO was strictly maintained at 0.1% (*v*/*v*) to exclude solvent-induced cytotoxicity.

Ac-YVAD-CMK, a selective Caspase-1 inhibitor, was included as a positive control and strongly suppressed IL-1β secretion, confirming the Caspase-1-dependent nature of the PM_2.5_-induced inflammatory response (Figure 3b). Importantly, the observation that the reduction in IL-1β release was due to the inhibitory effect of Sch B rather than a decrease in total cell number is consistent with the high cell viability shown in Figure 3a.

These results indicate that Sch B effectively attenuates PM_2.5_-induced inflammatory responses in THP-1 cells, with efficacy approaching that of the positive control.

#### 3.2.2. Sch B Reduces PM_2.5_-Induced Increase in Caspase-1 Activity

**Figure 4:**
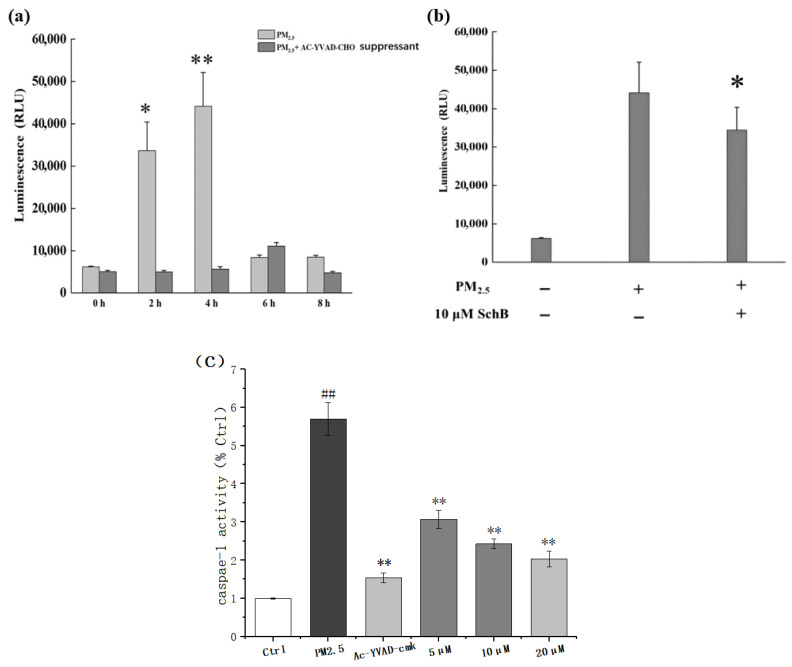
Sch B inhibits PM_2.5_-induced Caspase-1 activation in THP-1 cells. (**a**) Time-course of Caspase-1 activity following 100 μg/mL PM_2.5_ exposure. Ac-YVAD-CHO (1 μM kit-provided) was included during the luminescence reaction as a technical control to confirm assay specificity. (**b**) Effect of Sch B (10 μM) pretreatment on PM_2.5_-induced Caspase-1 activation. (**c**) Dose-dependent effect of Sch B (5, 10, and 20 μM) compared with the selective Caspase-1 inhibitor Ac-YVAD-CMK (10 μM, biological positive control). Data are presented as mean ± SD (*n* = 3). ## *p* < 0.01 vs. control; * *p* < 0.05 and ** *p* < 0.01 vs. the PM2.5-treated group.

Caspase-1 activity in THP-1 cells was measured at different time points following 100 μg/mL PM_2.5_ exposure. As shown in Figure 4a, Caspase-1 activity gradually increased and peaked at 4 h. To validate the model and ensure the specificity of the measured signal, Ac-YVAD-CHO (a kit-provided inhibitor) was used as a technical control during the assay phase. The results showed that PM_2.5_-induced luminescence was almost completely suppressed by the inclusion of Ac-YVAD-CHO, confirming that the detected enzymatic activity was specifically Caspase-1-dependent.

To assess the dose-dependent effect of Sch B, cells were pretreated with Sch B (5, 10, or 20 μM) or the biological positive control Ac-YVAD-CMK (10 μM). As shown in Figure 4c, PM_2.5_ markedly increased Caspase-1 activity relative to the blank control. Pretreatment with Sch B dose-dependently reduced this activity, with the 20 μM group reaching levels comparable to the Ac-YVAD-CMK positive control. These results collectively indicate that Sch B effectively attenuates PM_2.5_-induced Caspase-1 activation.

#### 3.2.3. Sch B Significantly Reduced the Increase in N-GSDMD

**Figure 5:**
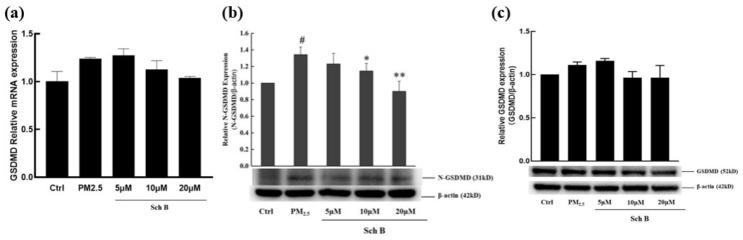
Sch B inhibits the cleavage of the pyroptosis effector protein GSDMD. (**a**) Results of different concentrations of Sch B on the transcription level of GSDMD. (**b**) Protein expression of N-GSDMD (~31 kDa) and representative Western blot bands. (**c**) Protein expression of full-length GSDMD (~52 kDa) and representative Western blot bands. These data suggest that Sch B reduces GSDMD-mediated pyroptosis signaling by suppressing the generation of N-GSDMD. Target protein levels were normalized to their respective β-actin (~42 kDa) controls to ensure comparability across different blots (* *p* < 0.05 vs. the group treated with 100 μg/mL PM_2.5_, ** *p* < 0.01 vs. the group treated with 100 μg/mL PM_2.5_; # *p* < 0.05 vs. control).

Evaluation of GSDMD mRNA levels showed that the levels of this transcript did not differ between the PM_2.5_-treated and blank control groups, and there were no significant changes in its expression following treatment with various concentrations of Sch B (Figure 5a). Consistent with the transcriptional data, the protein expression of full-length GSDMD (~53 kDa) showed no significant changes in response to the treatments (Figure 5c).

N-terminal fragment of GSDMD (N-GSDMD, ~31 kDa) level significantly increased in response to PM_2.5_ treatment compared with that in the control (Figure 5b), and this increase was attenuated in a concentration-dependent manner upon exposure to Sch B. Treatment with 10 or 20 μM Sch B significantly reduced the level of N-GSDMD. To ensure precise quantification, full-length GSDMD and N-GSDMD were detected using the same protein samples and normalized to the corresponding internal control (β-actin, ~42 kDa) probed on the same membrane. These results, based on three independent biological replicates (*n* = 3), indicate that Sch B prevents PM_2.5_-induced pyroptosis by inhibiting GSDMD cleavage rather than affecting its total expression.

### 3.3. Sch B Is Associated with ESCRT-III–Related Membrane Repair Responses

#### 3.3.1. Sch B Promotes Intracellular Ca^2+^ Influx

The plasma membrane serves as a vital semi-permeable barrier that maintains cellular homeostasis by separating the cell from its environment. Under pathological conditions, such as exposure to pore-forming proteins or toxins, membrane integrity is compromised. To counteract these lethal threats, cells have evolved sophisticated active repair mechanisms, and a rapid influx of extracellular Ca^2+^ is the pivotal core signal that triggers these plasma membrane repair programs [13].

**Figure 6:**
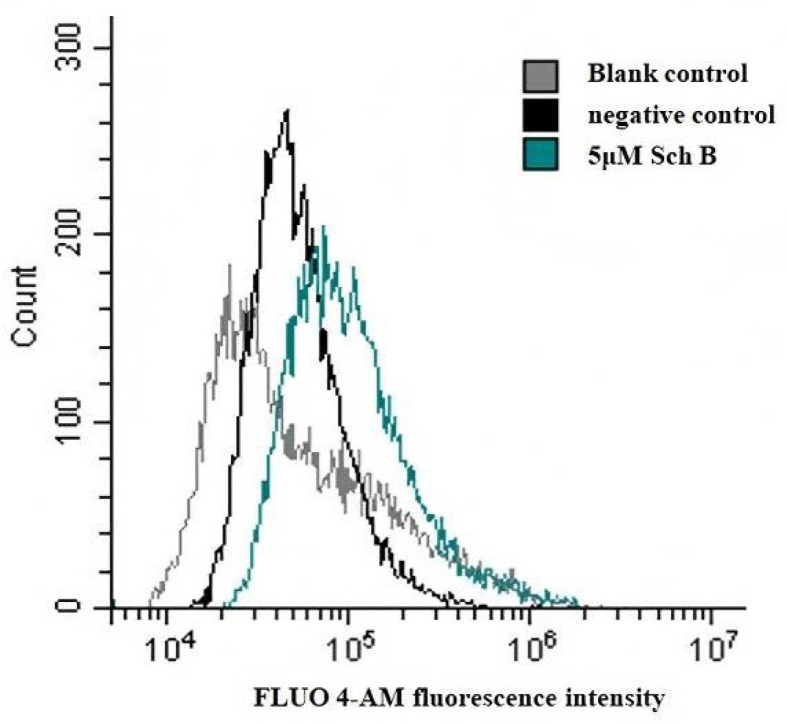
The effect of Sch B on the influx of Ca^2+^. In the PM_2.5_-THP-1 cell injury model, 5 μM Sch B can promote Ca^2+^ influx. Representative flow cytometry histograms (Fluo-4 AM) showing intracellular Ca^2+^ levels in THP-1 cells. While PM_2.5_ causes baseline membrane stress, 5 μM Sch B significantly enhances Ca^2+^ influx. This regulated increase acts as a biological trigger for downstream membrane repair mechanisms (e.g., lysosomal exocytosis or ESCRT assembly) to counteract PM_2.5_-induced pyroptotic pore formation.

Next, we evaluated the effect of 5 μM Sch B on PM_2.5_-stimulated THP-1 cell pyroptosis (Figure 6 Our data demonstrated that pretreatment with 5 μM Sch B further promoted Ca^2+^ influx compared with both the blank control and the PM_2.5_-treated negative control groups. Importantly, since 5 μM Sch B was previously confirmed to be non-cytotoxic in our viability assays, this observed increase in Ca^2+^ is unlikely to represent passive leakage caused by aggravated damage. Instead, it likely represents a regulated compensatory response. This finding is consistent with research by Scheffer L. et al., which reported that a transient increase in Ca^2+^ triggered by membrane injury leads to the recruitment and assembly of repair machinery, such as ESCRT-III and accessory proteins, at the site of damage. Therefore, our results suggest that Sch B may mitigate PM_2.5_-induced injury by enhancing Ca^2+^ dependent signaling pathways that facilitate rapid membrane resealing, thereby protecting the cells from GSDMD-mediated perforation [13,14,15].

#### 3.3.2. Effects of Sch B on the Expression of ESCRT-Associated Markers

**Figure 7:**
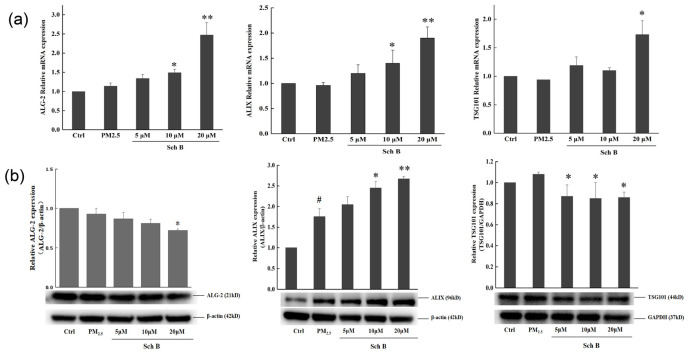
(**a**) Effects of different concentrations of Sch B on the transcript levels of ALG-2, ALIX, and TSG101. (**b**) Effects of different concentrations of Sch B on the expression levels of TSG101, ALIX, and ALG-2 (* *p* < 0.05 and ** *p* < 0.01 vs. 100 μg/mL PM_2.5_-treated group; # *p* < 0.05 vs. control group).

Real-time PCR results showed that ALG-2, ALIX, and TSG101 mRNA expression levels were the same in PM_2.5_-treated cells compared with the blank control; however, Sch B induced a concentration-dependent increase in their expression, with the most significant increase in expression at 10 and 20 μM Sch B. The expression of ALG-2 and TSG101 in the cytoplasm was also consistent in PM_2.5_-treated cells and in blank control cells Figure 7a).

At the protein level (Figure 7b), the expression of ALIX was significantly up-regulated in PM_2.5_-treated cells compared to the blank control, suggesting that endogenous self-repair of the cell membrane is initiated after PM_2.5_-induced damage. Pretreatment with 10 μM and 20 μM Sch B further increased ALIX expression, consistent with the transcriptional data. In contrast, the total protein expression of ALG-2 and TSG101 remained relatively stable across groups. While their total expression was consistent, the up-regulation of their transcripts and the significant increase in ALIX suggest that Sch B strengthens the molecular reservoir of the ESCRT system, potentially facilitating the assembly of repair complexes as suggested by the associated literature [16,17].

## 4. Discussion

Pyroptosis is a form of programmed cell death characterized by cell swelling, plasma membrane rupture, and the subsequent release of intracellular contents, which leads to a robust inflammatory response. This process has been implicated in the pathogenesis of infectious diseases, autoimmune disorders, cardiovascular diseases, cancers, and various inflammatory conditions [18,19,20,21]. Recent studies have further demonstrated that airborne particulate matter, including PM_2.5_, can upregulate key pyroptosis-related markers such as NLRP3, Caspase-1, IL-1β, and IL-18 in cellular models, thereby promoting inflammatory cell death [22,23]. Environmental particulate matter exposure is increasingly recognized as a trigger of inflammatory diseases, including respiratory inflammation, cardiovascular disorders, and skin inflammation. PM_2.5_-induced activation of inflammasome pathways and pyroptosis has been implicated in the pathogenesis of these conditions.

*Schisandra chinensis* has long been used in traditional medicine for the treatment of respiratory and fatigue-related disorders. Modern pharmacological studies have demonstrated that its major lignans, particularly Sch B, possess anti-inflammatory and cytoprotective properties. partly through the inhibition of NF-κB and MAPK signaling pathways [24].The present findings extend these pharmacological observations by demonstrating that Sch B suppresses Caspase-1-mediated pyroptosis and modulates ESCRT-associated markers in a PM_2.5_-induced cellular injury model This highlights a novel facet of Sch B’s cytoprotective potential beyond general anti-inflammatory signaling.

Consistent with these findings, PM_2.5_ has been reported to induce pyroptosis through the cleavage of the pore-forming effector protein gasdermin D (GSDMD) into its active N-terminal fragment (N-GSDMD) [25]. The formation of N-GSDMD pores compromises plasma membrane integrity, facilitates the release of inflammatory cytokines such as IL-1β, and ultimately drives the progression of pyroptosis-associated inflammation. In the present study, PM_2.5_ stimulation significantly increased N-GSDMD levels and IL-1β release in THP-1 cells, confirming the establishment of a PM_2.5_-induced pyroptotic cell injury model.

Importantly, our results showed that Sch B did not significantly alter the transcriptional or total protein expression levels of full-length GSDMD but markedly reduced the generation of N-GSDMD. This finding suggests that Sch B attenuates PM_2.5_-induced pyroptosis primarily by inhibiting GSDMD cleavage/activation rather than suppressing basal GSDMD expression, thereby limiting membrane perforation. The inhibitory effect of Sch B on PM_2.5_-induced Caspase-1 activation was consistent with the effect observed with the selective Caspase-1 inhibitor.

The integrity of the plasma membrane is essential for maintaining cellular homeostasis and viability. Under physiological and pathological conditions, cells are frequently exposed to membrane-disrupting stresses, including pore-forming proteins and toxins. To counteract such damage, cells have evolved rapid plasma membrane repair mechanisms, many of which are initiated by an influx of extracellular Ca^2+^ ions [14,15,26]. In this study, Sch B treatment was associated with increased intracellular Ca^2+^ levels in PM_2.5_-stimulated THP-1 cells. While Ca^2+^-influx can reflect membrane damage, we interpret this increase as a potential signaling trigger for membrane repair. This is supported by the fact that the 5 μM concentration of Sch B was non-cytotoxic in our viability assays, suggesting a regulated compensatory response rather than passive leakage. However, the dual nature of Ca^2+^ signaling in this context remains a point of ambiguity.

Previous studies have demonstrated that Ca^2+^-dependent recruitment of the ESCRT-III machinery plays a critical role in repairing plasma membrane damage during pyroptosis. Scheffer et al. (2014) reported that upon membrane injury, the Ca^2+^-binding protein ALG-2 interacts with the adaptor protein ALIX [13], thereby recruiting ESCRT-III components and Vps4 to damaged membrane sites and facilitating membrane scission and shedding of injured regions. ALG-2 has been proposed to act as an upstream initiator of ESCRT-mediated membrane repair, in part through the recruitment of TSG101, a core component of the ESCRT-I complex that is essential for subsequent ESCRT assembly and membrane remodeling [17,27].

In line with these reports, our data showed that PM_2.5_ exposure alone induced an endogenous increase in ALIX protein levels, suggesting a self-repair attempt by the cells. Sch B treatment induced a concentration-dependent upregulation of ALG-2, ALIX, and TSG101 mRNA. These observations suggest an association between Sch B and the up-regulation of ESCRT-associated markers. Without direct localization data, these results imply that Sch B may enhance the molecular reservoir of the ESCRT system, potentially supporting cellular capacity for membrane maintenance.

Taken together, our findings indicate that Sch B exerts a protective effect against PM_2.5_-induced pyroptotic cell injury in THP-1 cells. This protection appears to involve two complementary processes: inhibition of GSDMD cleavage/activation, and an association with enhanced ESCRT-III–related markers [17,25].These mechanisms jointly contribute to the attenuation of inflammatory cytokine release and the preservation of membrane integrity during PM_2.5_-induced cellular stress.

Despite the mechanistic insights provided by the present study, several limitations should be acknowledged. First, PM_2.5_ is a heterogeneous mixture composed of diverse organics, metals, and other constituents; the specific chemical constituents—such as heavy metals, polycyclic aromatic hydrocarbons (PAHs), and endotoxins—responsible for triggering pyroptosis were not identified in this study. The lack of a detailed chemical profile remains a significant methodological limitation, as variations in PM_2.5_ composition from different sources and seasons may influence the magnitude of the observed inflammatory and pyroptotic responses. Variations in PM_2.5_ sources and chemical composition may influence the magnitude and nature of the observed inflammatory responses, which limits the generalizability of the findings. Second, the PM_2.5_-induced injury model employed here represents an acute in vitro exposure in undifferentiated THP-1 cells, which cannot fully recapitulate the complexity of chronic environmental exposure or the multicellular interactions occurring in vivo. Therefore, caution is warranted when extrapolating these results to physiological or clinical settings, and the potential of Sch B should not be overstated.

Furthermore, we lack direct evidence of functional membrane resealing kinetics or the recruitment of ESCRT-III components to the plasma membrane. The use of Annexin V/PI staining alone also has limitations in exclusively defining pyroptotic cell death.

In addition, although Sch B was shown to modulate GSDMD cleavage and associate with ESCRT-related markers, the upstream signaling events linking PM_2.5_ exposure to inflammasome activation and membrane repair were not systematically investigated. Alternative mechanisms, such as the involvement of oxidative stress, mitochondrial dysfunction, or other inflammatory signaling pathways, may also contribute to the protective effects of Sch B and should be considered in future studies.

Given the increasing health burden associated with environmental pollution, the identification of natural compounds capable of mitigating PM_2.5_-induced cellular damage is of considerable pharmacological interest.

Future research should aim to characterize the specific bioactive components of PM_2.5_ responsible for inducing pyroptosis and to determine whether Sch B exerts differential effects against distinct particulate constituents. In addition, in vivo studies and chronic exposure models will be required to further validate the anti-inflammatory and cytoprotective potential of Sch B under physiologically relevant conditions. Taken together, our findings demonstrate that Sch B not only inhibits Caspase-1 activation and N-GSDMD generation but also up-regulates ESCRT-associated markers, thereby alleviating PM_2.5_-induced inflammatory cell death. This study highlights the potential of Sch B as a protective agent against while emphasizing the necessity for further functional and in vivo validation.

## 5. Conclusions

In this study, Sch B was shown to attenuate PM_2.5_-induced pyroptosis in THP-1 cells, which was associated with reduced Caspase-1 activity and decreased cleavage of the pyroptotic effector protein GSDMD into its active N-terminal fragment. In addition, Sch B treatment was associated with an increase in Ca^2+^ influx and the upregulation of ESCRT-related markers, which may contribute to membrane repair responses during PM_2.5_-induced cellular injury.

Furthermore, we established an in vitro model of PM_2.5_-induced inflammatory injury. Together, these findings demonstrate the protective activity of Sch B within this in vitro model and provide mechanistic insights into its effects against PM_2.5_-induced cellular stress. Our results suggest that Sch B warrants further investigation in more physiologically relevant systems to fully determine its potential as a protective agent.

## Figures and Tables

**Table 1:** Primer sequences of genes analyzed by quantitative real-time PCR.

Gene		Primer Sequence (5′ to 3′)
GSDMD	Forward	GGACAGGCAAAGATCGCAG
	Reverse	CACTCAGCGAGTACACATTCATT
ALG-2	Forward	GGCAGACTGCATCTTAGTCAAC
	Reverse	GGTCTATGTGAGACAGGGACTT
AlIX	Forward	ATCGCTGCTAAACATTACCAGTT
	Reverse	AGGGTCCCAACAGTATCTGGA
TSG101	Forward	GAGAGCCAGCTCAAGAAAATGG
	Reverse	TGAGGTTCATTAGTTCCCTGGA
CHMP4B	Forward	TGCAGAGGAGATTTCAACAGC
	Reverse	TGTTTCGGGTCCACTGATTTC

## Data Availability

The datasets used and/or analysed during the current study are available from the corresponding author on reasonable request.

## Supplementary Materials

The following supporting information can be downloaded at https://www.mdpi.com/article/10.3390/membranes16050173/s1, Figure S1: Scanning electron microscopy (SEM) images of PM2.5 particles; File S1: Original Western Blot images (Supplementary_Material_Original_WB_Blots); File S2: Certificate of Analysis (COA) for PM2.5 (B21327_KB380140-COA.pdf).

## References

[1] Panossian A., Wikman G. (2008). Pharmacology of Schisandra chinensis Bail.: An overview of Russian research and uses in medicine. J. Ethnopharmacol..

[2] Luk K.F., Ko K.M., Ng K.M. (2008). Separation and purification of schisandrin B from Fructus Schisandrae. Ind. Eng. Chem. Res..

[3] Nasser M., Zhu S., Chen C., Zhao M., Huang H., Zhu P. (2020). A comprehensive review on schisandrin B and its biological properties. Oxidative Med. Cell. Longev..

[4] Zhang W., Sun Z., Meng F. (2017). Schisandrin B ameliorates myocardial ischemia/reperfusion injury through attenuation of endoplasmic reticulum stress-induced apoptosis. Inflammation.

[5] Guo M., An F., Yu H., Wei X., Hong M., Lu Y. (2017). Comparative effects of schisandrin A, B, and C on Propionibacterium acnes-induced, NLRP3 inflammasome activation-mediated IL-1β secretion and pyroptosis. Biomed. Pharmacother..

[6] Shou Y., Huang Y., Zhu X., Liu C., Hu Y., Wang H. (2019). A review of the possible associations between ambient PM2.5 exposures and the development of Alzheimer’s disease. Ecotoxicol. Environ. Saf..

[7] Niu L., Li L., Xing C., Luo B., Hu C., Song M., Niu J., Ruan Y., Sun X., Lei Y. (2021). Airborne particulate matter (PM2.5) triggers cornea inflammation and pyroptosis via NLRP3 activation. Ecotoxicol. Environ. Saf..

[8] Dong Y.-m., Liao L.-y., Li L., Yi F., Meng H., He Y.-f., Guo M.-m. (2019). Skin inflammation induced by ambient particulate matter in China. Sci. Total Environ..

[9] Duan S., Wang N., Huang L., Zhao Y., Shao H., Jin Y., Zhang R., Li C., Wu W., Wang J. (2019). NLRP3 inflammasome activation is associated with PM2. 5-induced cardiac functional and pathological injury in mice. Environ. Toxicol..

[10] Shukla S., Larsen K.P., Ou C., Rose K., Hurley J.H. (2022). In vitro reconstitution of calcium-dependent recruitment of the human ESCRT machinery in lysosomal membrane repair. Proc. Natl. Acad. Sci. USA.

[11] Ballweg K., Mutze K., Königshoff M., Eickelberg O., Meiners S. (2014). Cigarette smoke extract affects mitochondrial function in alveolar epithelial cells. Am. J. Physiol.-Lung Cell. Mol. Physiol..

[12] Heinrich M., Jalil B., Abdel-Tawab M., Echeverria J., Kulić Ž., McGaw L.J., Pezzuto J.M., Potterat O., Wang J.-B. (2022). Best practice in the chemical characterisation of extracts used in pharmacological and toxicological research—The ConPhyMP—Guidelines. Front. Pharmacol..

[13] Scheffer L.L., Sreetama S.C., Sharma N., Medikayala S., Brown K.J., Defour A., Jaiswal J.K. (2014). Mechanism of Ca^2+^-triggered ESCRT assembly and regulation of cell membrane repair. Nat. Commun..

[14] Espiritu R.A. (2021). Repairing plasma membrane damage in regulated necrotic cell death. Mol. Biol. Rep..

[15] Cheng X., Zhang X., Yu L., Xu H. (2015). Calcium signaling in membrane repair. Semin. Cell Dev. Biol..

[16] Sønder S.L., Boye T.L., Tölle R., Dengjel J., Maeda K., Jäättelä M., Simonsen A.C., Jaiswal J.K., Nylandsted J. (2019). Annexin A7 is required for ESCRT III-mediated plasma membrane repair. Sci. Rep..

[17] Rühl S., Shkarina K., Demarco B., Heilig R., Santos J.C., Broz P. (2018). ESCRT-dependent membrane repair negatively regulates pyroptosis downstream of GSDMD activation. Science.

[18] Shen H.-H., Yang Y.-X., Meng X., Luo X.-Y., Li X.-M., Shuai Z.-W., Ye D.-Q., Pan H.-F. (2018). NLRP3: A promising therapeutic target for autoimmune diseases. Autoimmun. Rev..

[19] Zhaolin Z., Guohua L., Shiyuan W., Zuo W. (2019). Role of pyroptosis in cardiovascular disease. Cell Prolif..

[20] Mangan M.S., Olhava E.J., Roush W.R., Seidel H.M., Glick G.D., Latz E. (2018). Targeting the NLRP3 inflammasome in inflammatory diseases. Nat. Rev. Drug Discov..

[21] Mamik M.K., Power C. (2017). Inflammasomes in neurological diseases: Emerging pathogenic and therapeutic concepts. Brain.

[22] Yang R., Yu H., Chen J., Zhu J., Song C., Zhou L., Sun Y., Zhang Q. (2021). Limonin attenuates LPS-induced hepatotoxicity by inhibiting pyroptosis via NLRP3/gasdermin D signaling pathway. J. Agric. Food Chem..

[23] Zhao M., Dai Y., Li P., Wang J., Ma T., Xu S. (2021). Inhibition of NLRP3 inflammasome activation and pyroptosis with the ethyl acetate fraction of Bungeanum ameliorated cognitive dysfunction in aged mice. Food Funct..

[24] Wu Y., Li Z.-c., Yao L.-q., Li M., Tang M. (2019). Schisandrin B alleviates acute oxidative stress via modulation of the Nrf2/Keap1-mediated antioxidant pathway. Appl. Physiol. Nutr. Metab..

[25] Kayagaki N., Stowe I.B., Lee B.L., O’Rourke K., Anderson K., Warming S., Cuellar T., Haley B., Roose-Girma M., Phung Q.T. (2015). Caspase-11 cleaves gasdermin D for non-canonical inflammasome signalling. Nature.

[26] Reddy A., Caler E.V., Andrews N.W. (2001). Plasma membrane repair is mediated by Ca^2+^-regulated exocytosis of lysosomes. Cell.

[27] Jimenez A.J., Maiuri P., Lafaurie-Janvore J., Divoux S., Piel M., Perez F. (2014). ESCRT machinery is required for plasma membrane repair. Science.

