# Supplementary material for: Schisandrin B Attenuates PM2.5-Induced Pyroptosis via Caspase-1 Inhibition and Membrane Repair
Source: Membranes (Basel). 2026 May 9;16(5):173. doi: 10.3390/membranes16050173 (PMC13209075; doi:10.3390/membranes16050173)
Supplement: Supplementary file 1 [file membranes-16-00173-s001.zip › B21327_KB380140-COA.pdf]

Shanghai yuanye Bio-Technology Co., Ltd

Shanghai yuanye Bio-Technology Co., Ltd
Tel: 400-666-5481 Fax: +86-21-55068248
Website: www.shyuanye.com
Email: 3008007435@qq.com

CERTIFICATE OF ANALYSIS

English Name：Schizandrin B

Molecular Formula：C23H28O6

Molecular Wt：400.471

CAS：61281-37-6

Storage Condition：2-8℃

Item No：B21327

Lot. Number：KB380140

Date of Testing：2025-05-23

Date of Retesting：2028-05-22

SPECIFICATION PROPERTIES	STANDARD	RESULTS
Appearance	White crystalline powder	White crystalline powder
Purity	98% min(HPLC)	98.5%

源叶®

Quality Assurance：Xiaoyong Tan

上海源叶生物科技有限公司
质检专用章

Quality Control：Yunfei Zhu
