# Supplementary material for: Schisandrin B Attenuates PM2.5-Induced Pyroptosis via Caspase-1 Inhibition and Membrane Repair
Source: Membranes (Basel). 2026 May 9;16(5):173. doi: 10.3390/membranes16050173 (PMC13209075; doi:10.3390/membranes16050173)

Full Uncropped Blots for N-GSDMD (Related to Figure 5b)

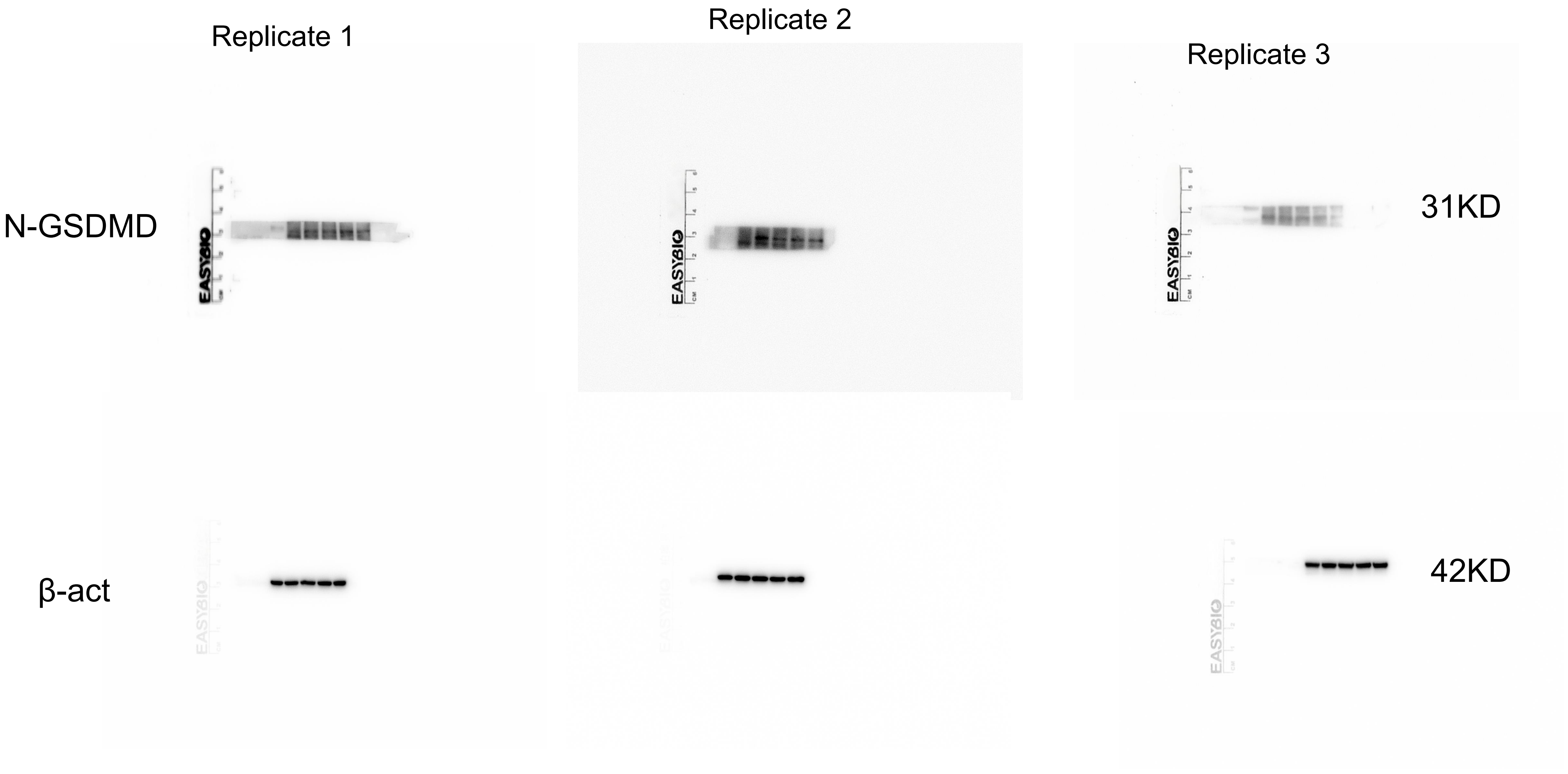

Full Uncropped Blots for GSDMD (Related to Figure 5c)

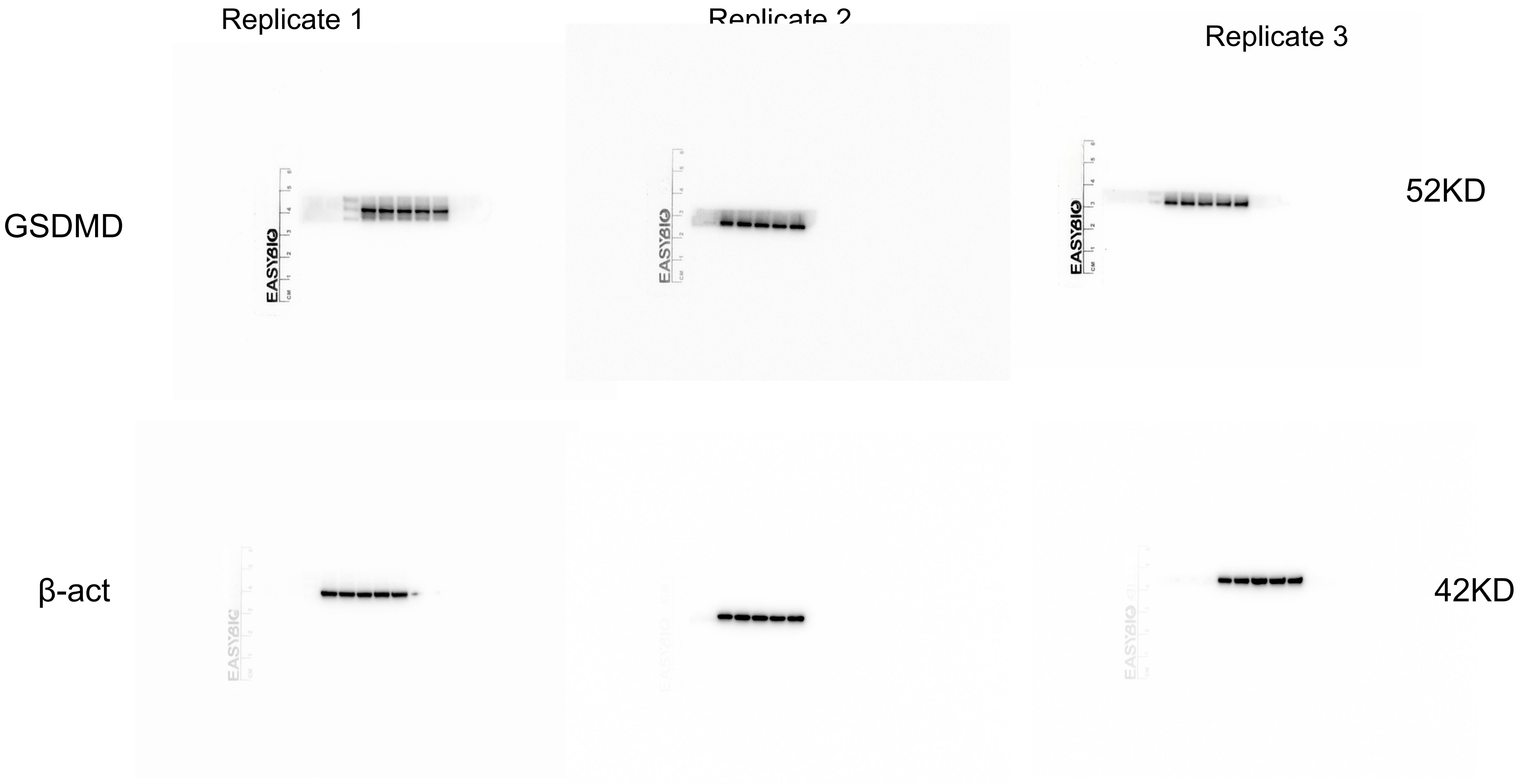

Full Uncropped Blots for ALG-2(Related to Figure 7b)

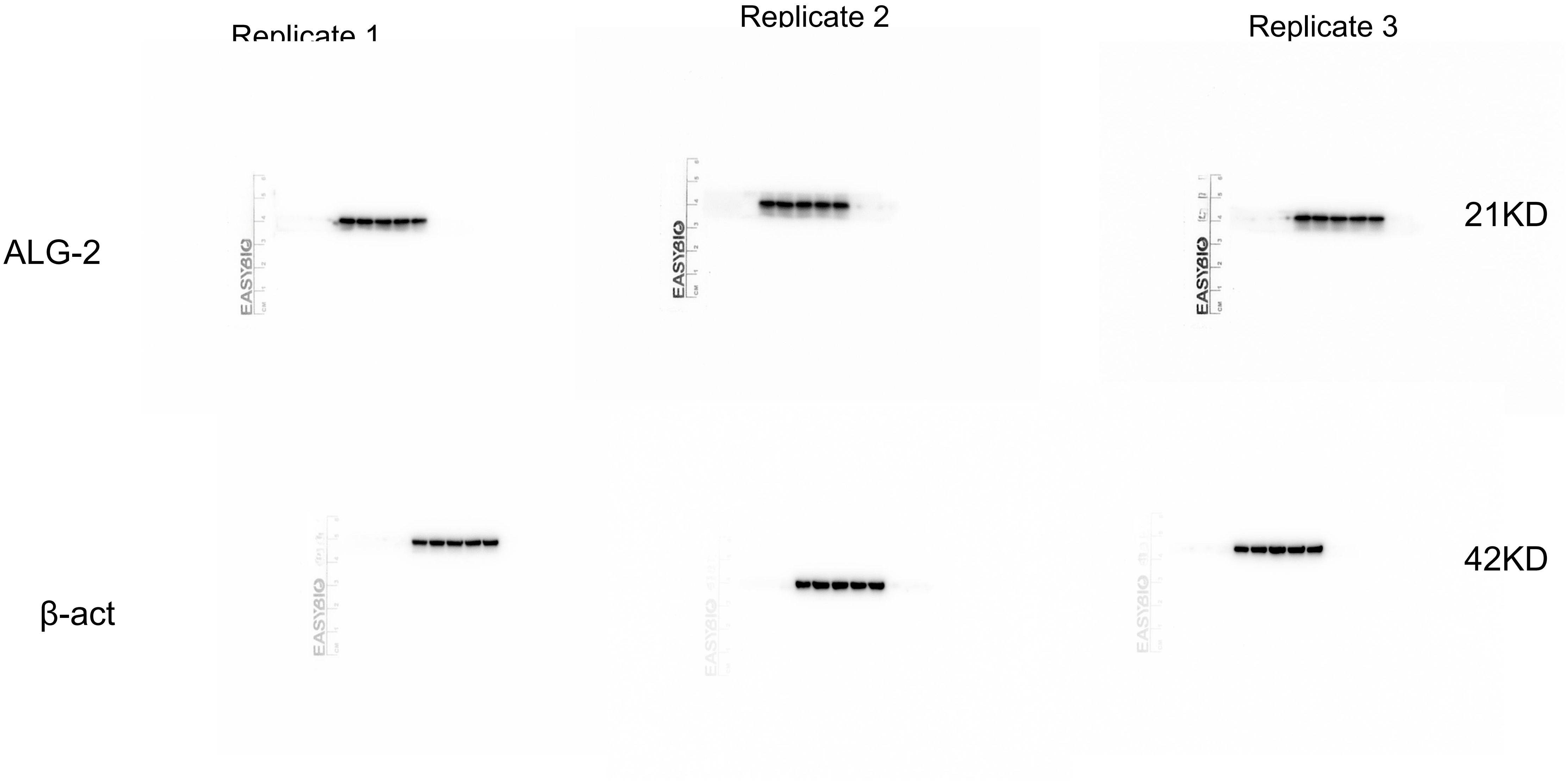

Full Uncropped Blots for ALIX(Related to Figure 7b)

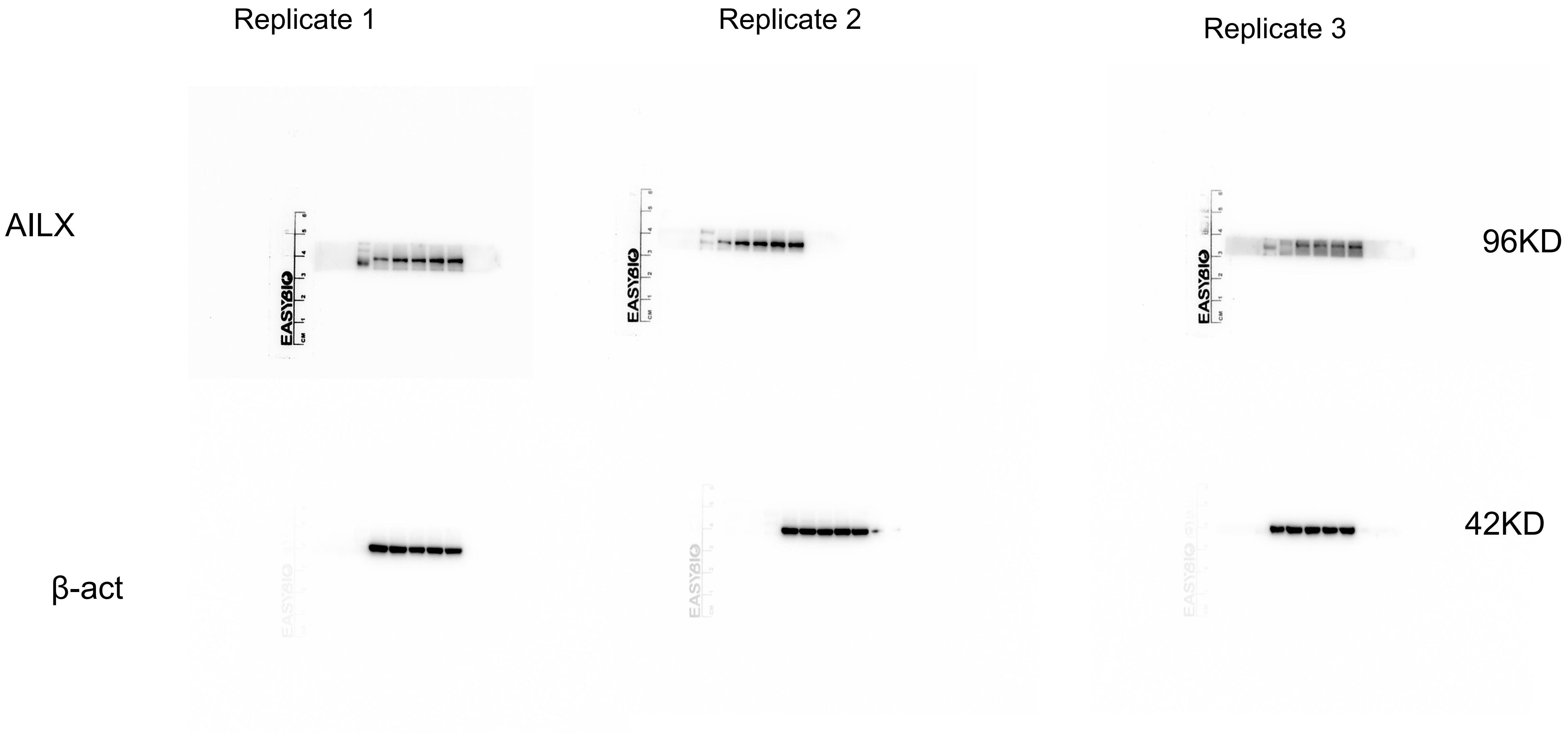

Full Uncropped Blots forTSG101(Related to Figure 7b)

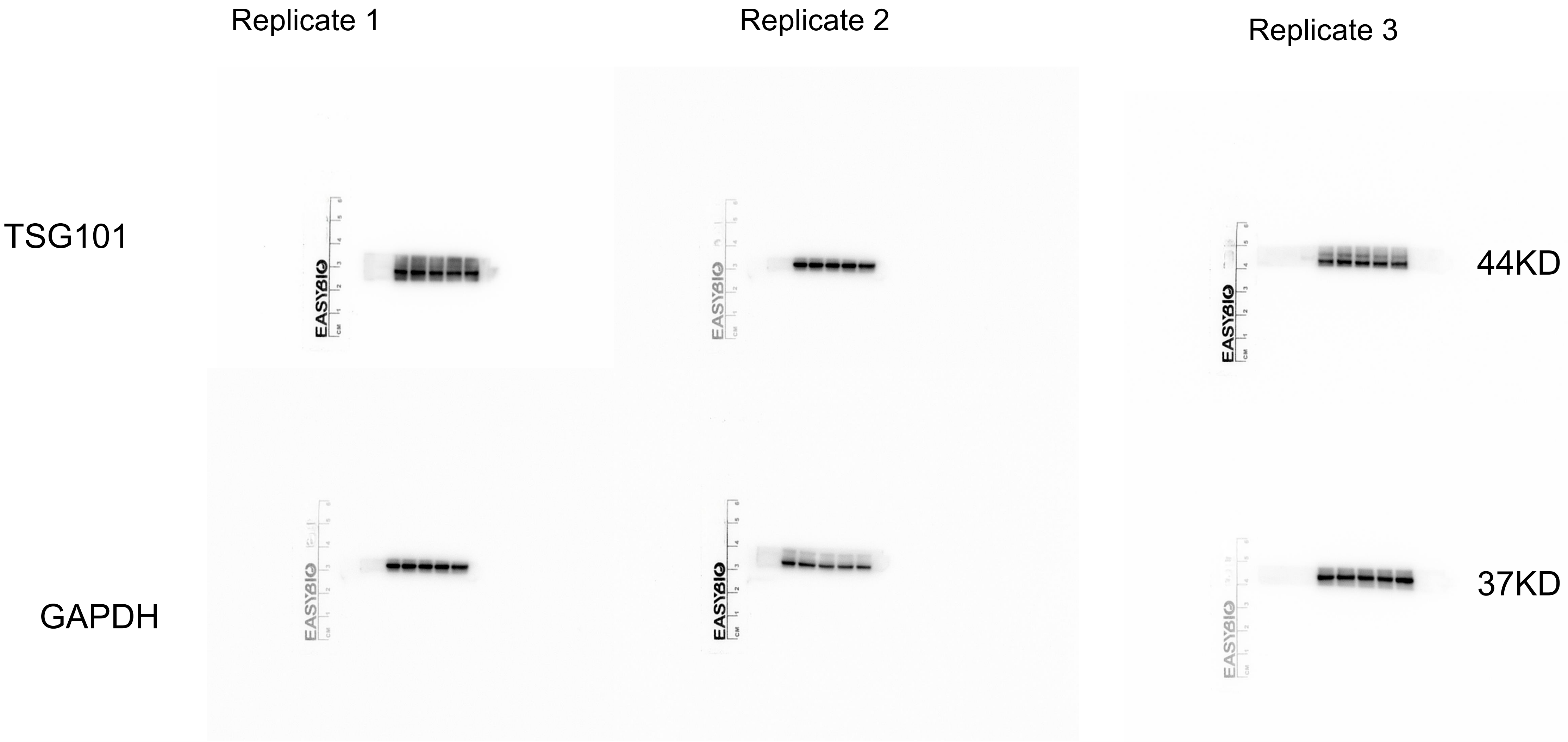

Supplement: Supplementary file 1 [file membranes-16-00173-s001.zip › Supplementary_Material_Original_WB_Blots.pdf]
