# Supplementary figures and images for: Schisandrin B Attenuates PM2.5-Induced Pyroptosis via Caspase-1 Inhibition and Membrane Repair
Source: Membranes (Basel). 2026 May 9;16(5):173. doi: 10.3390/membranes16050173 (PMC13209075; doi:10.3390/membranes16050173)

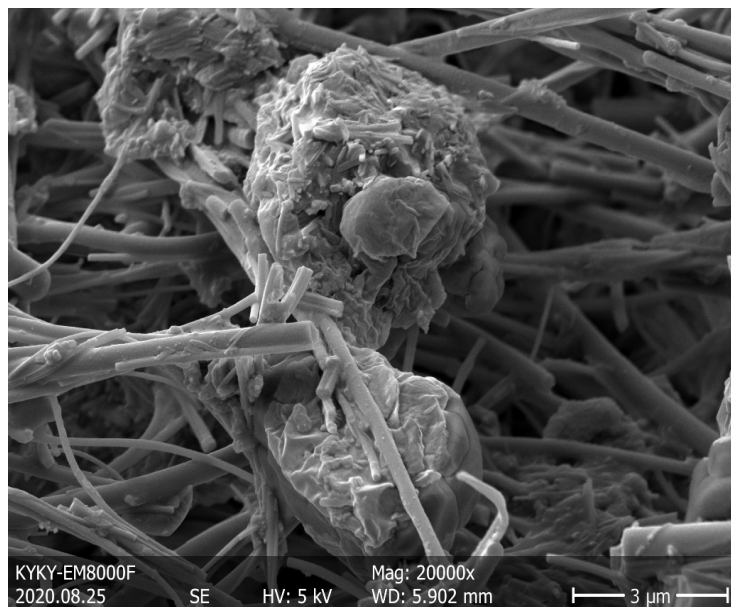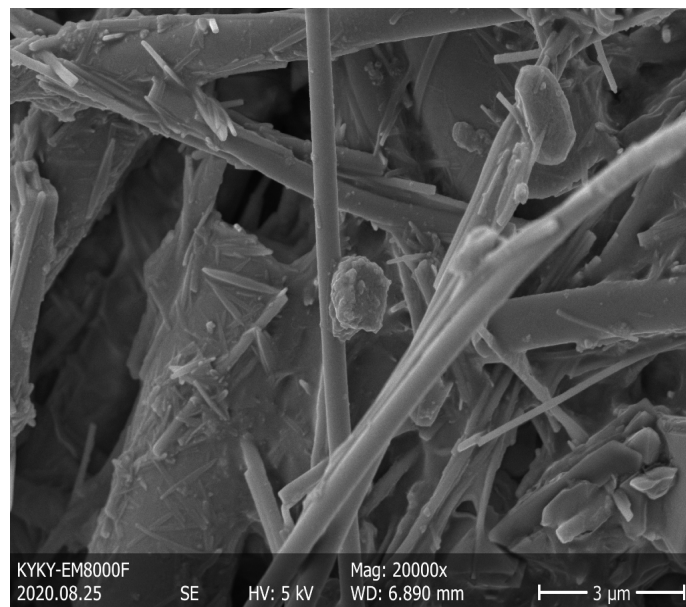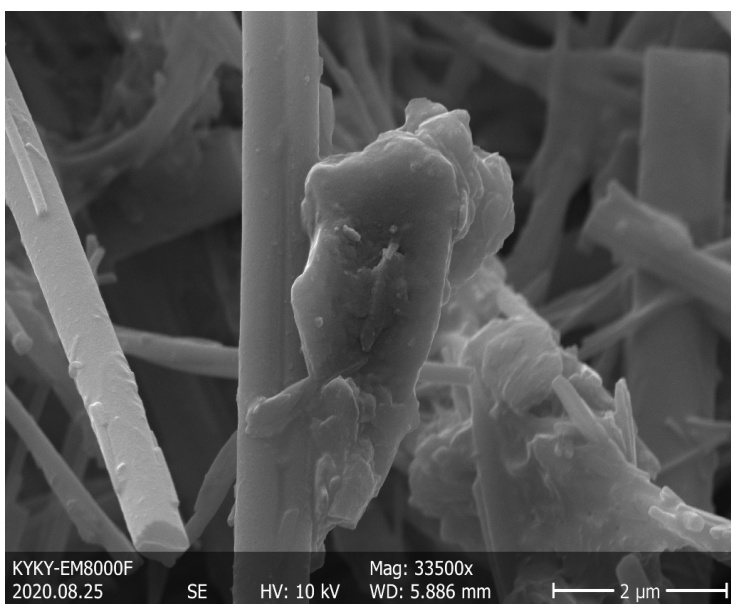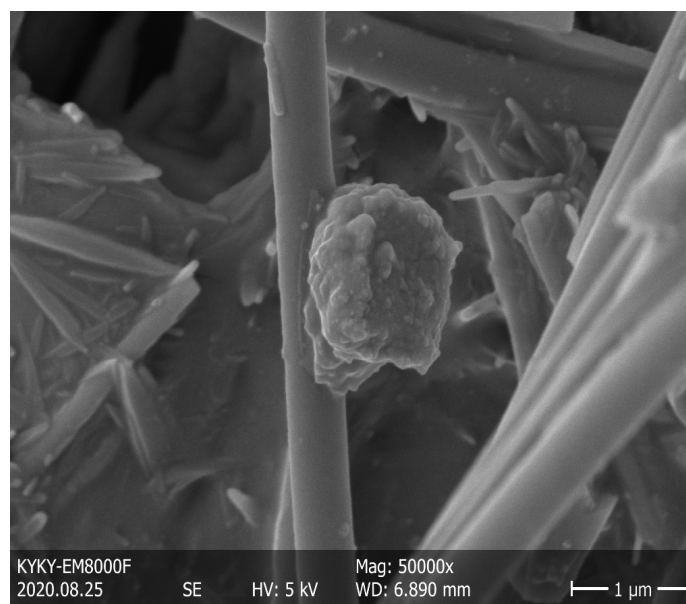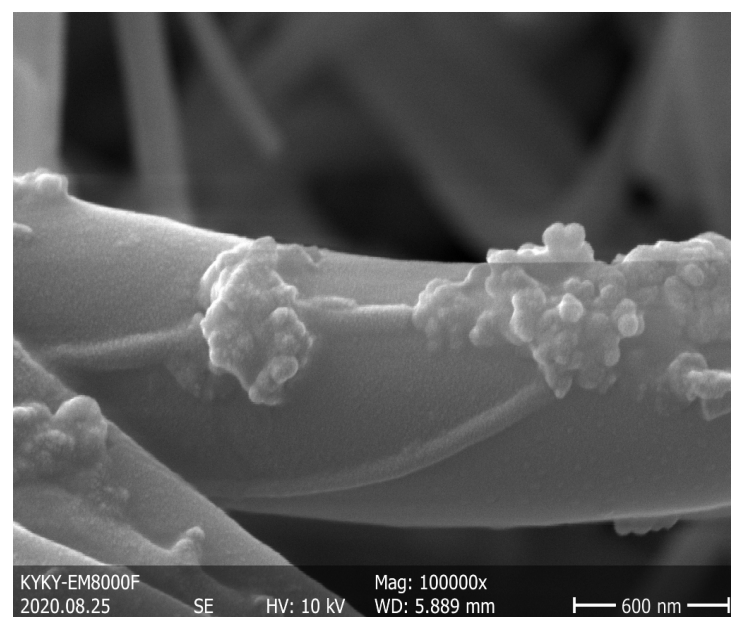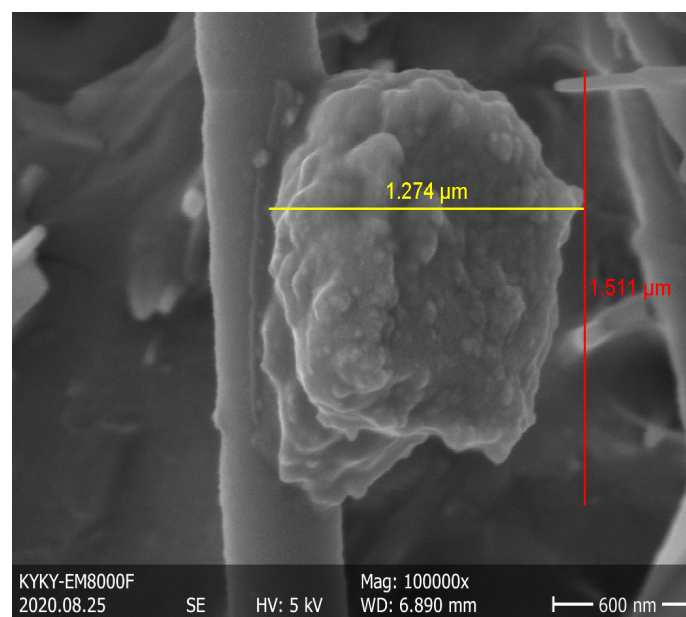

Supplement: Supplementary file 1 [file membranes-16-00173-s001.zip › Supplemengtary FigureS1.pdf]
